# Human Alzheimer’s disease reactive astrocytes exhibit a loss of homeostastic gene expression

**DOI:** 10.1186/s40478-023-01624-8

**Published:** 2023-08-02

**Authors:** David L. Dai, Mingyao Li, Edward B. Lee

**Affiliations:** 1 Translational Neuropathology Research Laboratory, Department of Pathology and Laboratory Medicine, Perelman School of Medicine, University of Pennsylvania, Philadelphia, 19104 USA; 2 Department of Biostatistics, Epidemiology, and Informatics, Perelman School of Medicine, University of Pennsylvania, Philadelphia, 19104 USA

## Abstract

Astrocytes are one of the brain’s major cell types and are responsible for maintaining neuronal homeostasis via regulating the extracellular environment, providing metabolic support, and modulating synaptic activity. In neurodegenerative diseases, such as Alzheimer’s disease, astrocytes can take on a hypertrophic appearance. These reactive astrocytes are canonically associated with increases in cytoskeletal proteins, such as glial fibrillary acidic protein and vimentin. However, the molecular alterations that characterize astrocytes in human disease tissues have not been extensively studied with single cell resolution. Using single nucleus RNA sequencing data from normal, pathologic aging, and Alzheimer’s disease brains, we identified the transcriptomic changes associated with reactive astrocytes. Deep learning-based clustering algorithms denoised expression data for 17,012 genes and clustered 15,529 astrocyte nuclei, identifying protoplasmic, gray matter and fibrous, white matter astrocyte clusters. RNA trajectory analyses revealed a spectrum of reactivity within protoplasmic astrocytes characterized by a modest increase of reactive genes and a marked decrease in homeostatic genes. Amyloid but not tau pathology correlated with astrocyte reactivity. To identify reactivity-associated genes, linear regressions of gene expression versus reactivity were used to identify the top 52 upregulated and 144 downregulated genes. Gene Ontology analysis revealed that upregulated genes were associated with cellular growth, responses to metal ions, inflammation, and proteostasis. Downregulated genes were involved in cellular interactions, neuronal development, ERBB signaling, and synapse regulation. Transcription factors were significantly enriched among the downregulated genes. Using co-immunofluorescence staining of Alzheimer’s disease brain tissues, we confirmed pathologic downregulation of ERBB4 and transcription factor NFIA in reactive astrocytes. Our findings reveal that protoplasmic, gray matter astrocytes in Alzheimer’s disease exist within a spectrum of reactivity that is marked by a strong loss of normal function.

**Supplementary Information:**

The online version contains supplementary material available at 10.1186/s40478-023-01624-8.

## Introduction

Alzheimer’s disease is the most common neurodegenerative disease and the leading cause of dementia. Neuropathologically, Alzheimer’s disease is characterized by the accumulation of beta-amyloid plaques and tau neurofibrillary tangles.

Astrocytes are one of the brain’s major cell types, composing ~ 10% of all brain cells [54]. They are crucial for maintaining neuronal homeostasis via regulating the extracellular environment, regulating blood flow, providing metabolic support, maintaining the blood–brain-barrier, and axon guidance [70]. Notably, astrocytes are structurally and functionally attuned to their local environments. In healthy brains, protoplasmic astrocytes found in cortical gray matter are globoid in shape with short processes and are specialized for uptaking neurotransmitters, propagating calcium signals through gap junctions, and forming one component of tripartite synapses. In contrast, fibrous astrocytes in white matter have elongated cytoskeletons and processes which play important roles in myelination and ion buffering at nodes of Ranvier [7, 45].

In many neurodegenerative diseases, including Alzheimer’s disease, astrocytes display altered morphologies. These pathologic, or reactive, glia are canonically associated with increases in cytoskeletal proteins, such as glial fibrillary acidic protein (GFAP) and vimentin (VIM) [25]. Attempts to study reactive astrocytes in mice led to characterizations of neurotoxic A1 and neuroprotective A2 astrocytes [43, 80], but these designations have been shown to be incomplete and overly simplistic. Furthermore, there is sometimes a poor concordance of gene expression patterns between mouse and human reactive astrocytes [16], emphasizing the need to characterize reactive astrocyte changes in humans.

In this study, we analyzed a single nucleus RNA sequencing dataset of normal, pathological aging, and Alzheimer’s disease brains [50] to investigate how human astrocytes are transcriptomically changed in neurodegenerative disease. We use novel bioinformatics approaches to identify a spectrum of reactivity within protoplasmic, gray matter astrocytes. We observe that astrocyte reactivity is associated with amyloid plaque deposition but not tauopathy. We also observed that human reactive astrocyte transcriptome is characterized by a marked downregulation of homeostatic genes enriched for transcription factors, suggesting that the loss of homeostatic function in reactive astrocytes may contribute to Alzheimer’s disease.

## Methods

### Isolating sequenced astrocytes

122,606 nuclei from the dorsolateral pre-frontal cortex of 15 samples that varied by amyloid and tau pathology as well as *APOE* and *TREM2* genotypes (Additional file 1: Table S1) were previously sequenced, clustered, and annotated using cell type-specific marker genes as described previously [50].

In brief, gray matter and underlying white matter from donors’ middle frontal gyrus (Broadmann area 9) was dounce homogenized in buffer composed of 0.25 M sucrose in TKM [50 mM Tris–HCl, pH 7.5, 25 mM KCl, 14 mM MgCl_2_, and 0.4 U/uL RNAse inhibitor (Promega)]. Homogenate was mixed with 2.3 M sucrose + TKM to adjust sucrose concentration to 1.6 M. Homogenate was overlayed on 3 mL of 1.8 M sucrose + TKM cushion and centrifuged at 100,000×*g* for 45 min at 4 °C (Sw-41Ti rotor in an XPN-80 ultracentrifuge). Supernatant was removed, and pelleted nuclei were re-suspended in PBS with RNAse inhibitor. Nuclei were sequenced by the Center for Applied Genomics Sequencing Core at the Children’s Hospital of Philadelphia using 10 × Genomics’ Chromium 3’ Single Cell Sequencing system to generate libraries and an Illumina HiSeq 2500 for sequencing. Reads were aligned to the human genome. Nuclei expressing 200–4000 genes and with < 5% of UMIs attributed to mitochondrial genes were iteratively clustered and annotated using cell type-specific marker genes to identify 122,606 nuclei that consistently belonged to the same cell type-specific cluster.

For each sequenced tissue sample, adjacent tissue was also fixed in formalin, stained for neuropathology, and rated based on pathological features as described previously [50]. Briefly, tissue sections were separately stained for beta-amyloid (NAB228) and phospho-tau (PHF1). Sections were assessed by two independent neuropathologists. Designations of A + were given to samples with amyloid plaques, and designations of T + were given to samples with neurofibrillary tangles with or without neuropil threads. The presence of tau-positive plaque-associated neuronal dystrophies was used to inform the A score. These ratings were compiled into an AT-score for each sample (Additional file 1: Table S1).

15,683 astrocyte nuclei were subset for further analysis.

### Clustering astrocytes and denoising gene expression

The astrocyte dataset encompassing 15,683 nuclei and 26,423 genes were filtered for genes expressed in less than 30 nuclei (< 0.2% of dataset) and nuclei expressing fewer than 2500 genes to respectively eliminate lowly expressed genes that increase statistical noise and data that may be derived from the artifactual sequencing of two nuclei together (doublet) that can skew clustering and gene expression denoising. CarDEC was used to analyze the resulting 15,529 nuclei × 17,012 genes for cell type clustering and gene expression denoising [37]. CarDEC is a deep learning algorithm that iteratively clusters nuclei and denoises gene expression to enable accurate clustering and robust gene expression data for downstream analyses. Nuclei were iteratively clustered at differing resolutions to identify the clustering resolution most appropriate for downstream analyses. To visualize nuclei’s cluster assignments across an increasing number of clusters, the function *sankeyNetwork* from the networkD3 package [1] was used with each cluster assignment inputted as nodes and nuclei inputted as links.

### Identifying astrocyte populations

To annotate astrocyte clusters, differential gene expression (DGE) analysis was performed for each astrocyte cluster versus the rest of the astrocyte nuclei. DGE analysis was conducted on denoised, normalized, natural log-transformed count data using the Wilcoxon rank-sum method with Benjamini–Hochberg *p*-value correction method for multiple comparisons using Scanpy’s *rank_genes_groups* function [76]. Differentially expressed genes were annotated for cell type-specific expression using our full human AD single nucleus RNA sequencing (snRNAseq) dataset [50], an RNA sequencing dataset of acutely isolated human brain cell types [83], and manual literature search of genes’ expression profiles and functions. Two astrocyte clusters, green/3 and red/4, were respectively enriched for neuron and oligodendrocyte-specific genes and were annotated as astrocyte-neuron and astrocyte-oligodendrocyte doublets that likely resulted from sequencing two nuclei within the same droplet. True astrocyte clusters were annotated following a literature search of gene expression and functional differences between astrocyte subtypes, including protoplasmic and fibrous astrocytes. Feature plots of astrocyte nuclei colored by gene expression were generated using Scanpy’s [76] *pl.umap* function. Heatmaps of astrocyte nuclei colored by groups of cell type-specific marker genes were generated using Scanpy’s *pl.heatmap* function. At the four-cluster resolution, this resulted in 7153 protoplasmic astrocytes, 4184 fibrous astrocytes, 2161 astrocyte-neuron doublets, and 2031 astrocyte-oligodendrocyte doublets.

### Pseudotime trajectory analysis

Protoplasmic and fibrous astrocyte nuclei were replotted using partition-based graph abstraction (PAGA) [77], which generates topology-preserving maps of single cell RNA sequencing data. PAGA was initialized using Scanpy’s *tl.paga* function. The graph of single nuclei was generated using *tl.draw_graph* and plotted using *pl.draw_graph* with *init_pos* set to ‘paga’. Diffusion pseudotime was initialized using the *tl.dpt* function with a root nucleus set to the minimum of the FA1 axis, where the protoplasmic astrocyte cluster’s homeostatic gene expression was the highest and reactive astrocyte gene expression was lowest.

### Pseudotime correlations with pathology

Linear mixed effects modeling was conducted using the *lme* function from the nlme package [56]. Nuclei pseudotime was input as the dependent variable. The presence of amyloid pathology, tau pathology, *APOE* genotype, *TREM2* genotype, and sample’s age at death were input as fixed, independent variables. The sample ID that each nucleus was associated with was used as a random, independent variable. Amyloid and tau pathologies were input separately as binary “0” if the tissue the nucleus originated from did not have amyloid/tau pathology or “1” if it did have amyloid/tau pathology. For *APOE* and *TREM2* genotypes, the E3/E3 and WT alleles were respectively used as the reference level. All sequenced nuclei were from male brains, so sex was omitted.

$$\begin{aligned} & {\text{Fixed}} = {\text{Amyloid}} + {\text{Tau}} + TREM2 + APOE + {\text{ Age}}\;{\text{at}}\;{\text{Death}} \\ & {\text{Random}} = \;\sim 1\left| {{\text{sampleID}}} \right. \\ \end{aligned}$$

### Linear regressions of gene expression changes over pseudotime

Protoplasmic astrocyte nuclei pseudotime values obtained from PAGA were non-uniformly distributed from 0 to 1. To prevent skewing of the regression analysis due to a small subset of nuclei, we rank ordered nuclei pseudotime to generate a consistent distribution of nuclei across pseudotime. Linear regressions of nuclei’s ranked pseudotime versus denoised, normalized, and natural log-transformed gene expression were calculated for each gene using the Scipy package’s *stats.linregress* function [71]. Genes were thresholded for linear regression beta (effect size) > 0.1, R^2^ (variance) > 0.1, and *p*-value < 0.05 after Bonferroni correction for multiple comparisons to identify 196 significantly dysregulated genes that changed the most over pseudotime and whose change were the most explanatory over pseudotime. 52 of these genes were upregulated, while 144 were downregulated.

Linear regression data was used to generate MA-style plots. First, denoised gene expression was normalized and natural log-transformed using Scanpy’s *pp.normalize_total* and *pp.log1p* functions, respectively. Average gene expression was calculated by taking the mean average of each gene across all protoplasmic astrocyte nuclei. The first MA-style visualization consists of linear regression beta vs average expression. Data is also presented as linear regression beta versus R^2^ plots using the ggplot2 package [74]. *MALAT1*, a transcript known to exhibit very high average nuclear expression was removed from MA-style plots to improve visualization.

### Transcriptomic dysregulation comparisons with amyloid responsive microglia

2773 microglia nuclei from the same AD snRNAseq dataset were analyzed using the same methodology, namely denoising gene expression with CarDEC, normalizing and natural log-transforming gene expression using Scanpy, calculating each gene’s average expression, calculating pseudotime with PAGA, and generating linear regressions of gene expression changes. Prior to initiating CarDEC, genes expressed in fewer than 10 nuclei were removed using Scanpy’s *pp.filter_genes* function to remove lowly expressed genes that could skew gene expression denoising. *MALAT1* was again removed from MA-style plots.

### GO enrichment analysis

The 52 significantly upregulated and 144 downregulated genes were separately input into Metascape [86] with the total 17,012 genes expressed in astrocytes used as the reference transcriptome. Metascape draws gene ontology (GO) terms from over 40 independent databases and clusters similar GO terms into non-redundant groups enabling robust identification of non-overlapping enriched pathways. The top hits were then manually annotated into overarching themes.

### Comparisons to mouse astrocytes from AD models

Transcriptomic signatures of astrocytes from AD mouse models were obtained from recent studies [29, 32]. Mouse to human gene conversions were conducted using homology data available through the Mouse Genome Database [5]. snRNAseq of mouse brains identified a cluster of astrocytes that was enriched among 5xFAD mice, compared to wild-type [29]. These disease-associated astrocytes were characterized by 254 dysregulated genes (239 upregulated, 15 downregulated), of which 224 (210 upregulated, 14 downregulated) were successfully mapped to human genes in our dataset. Translating-ribosome-affinity purification followed by mRNA sequencing (TRAP-Seq) was recently performed on the cortex of late-stage (12 months) APP/PS1 mice as well as the cortex and spinal cord of late-stage (5 months) MAPT^P301S^ mice. After mapping to human genes in our dataset, APP/PS1 mice astrocytes expressed 10,087 genes and exhibited dysregulation of 2499 genes (1543 upregulated, 956 downregulated). After mapping to human genes in our dataset, MAPT^P301S^ mice astrocytes expressed 9429 genes in the cortex and exhibited dysregulations of 36 genes (20 upregulated, 16 downregulated), while 9427 genes were expressed in the spinal cord, and 1686 genes (870 upregulated, 816 downregulated) were dysregulated in the spinal cord. Dysregulated genes from these AD animal models were compared with our human reactive astrocyte transcriptome to identify transcriptomic differences in reactive astrocytes between human samples and AD animal models. Gene enrichment analysis between human and mouse dysregulated genes were performed using Fisher’s exact tests from Prism 9 software.

#### AD GWAS

Genes associated with AD risk development were obtained from a recent AD genome-wide association study (GWAS) meta-analysis, wherein 75 loci were identified to be associated with AD risk, which corresponded to 77 implicated nearest protein-coding genes [3]. AD GWAS hits were assessed for enrichment in astrocytes using differential gene expression analysis and mapped onto the human reactive astrocyte transcriptome to identify putative AD risk genes most dysregulated in reactive astrocytes.

#### Differential gene expression analysis

Differential gene expression analysis between astrocyte nuclei and other brain cell types was conducted on normalized, natural log-transformed count data using the Wilcoxon rank-sum method with Benjamini–Hochberg *p*-value correction method for multiple comparisons using Scanpy’s *rank_genes_groups* function. 8640 genes were enriched in astrocytes vs rest; these can be found in Additional file 2: Table S4.

#### Transcription factor enrichment among downregulated genes

A list of 1639 known and likely human transcription factors was obtained and compared to our dataset [38]. 1129 transcription factors were observed among the 17,012 genes in our dataset. Among the 196 most dysregulated genes, 18 were transcription factors; 1 of 52 upregulated genes was a transcription factor, and 17 of 144 downregulated genes were transcription factors. Chi-squared tests were performed to assess for transcription factor enrichment among gene lists.

#### Brain donor selection of validation autopsy cohort

Ten pathologically determined Alzheimer’s disease and neurologically normal brain donors were selected from the University of Pennsylvania Center for Neurodegenerative Disease Research (CNDR) Brain Bank for neuropathologic confirmation of bioinformatic findings. Alzheimer’s disease donors were selected based on the following criteria: clinical diagnoses of probable or possible Alzheimer’s disease as well as dementia of undetermined etiology; high or intermediate Alzheimer’s disease neuropathologic change (ADNC); age 65 or older at death; absence of autosomal dominant genetic variant such as *PSEN1*; and absence of *TREM2* genetic risk variants. For each donor, the middle frontal gyrus, angular gyrus, and superior/middle temporal gyrus were previously assessed for amyloid, tau, alpha-synuclein, and TDP-43 pathologies and sections were obtained for those regions with moderate (2+) tau, neuritic plaques, and amyloid pathologies as well as no or rare alpha-synuclein and TDP-43 pathology.

Neurologically normal control donors were selected based on the following criteria: clinical diagnoses of normal or depression; no or low ADNC without other frontal or temporal lobe proteinopathies, cerebrovascular disease, or hippocampal sclerosis; A, B, and C scores less than 3; age 65 or older at death; absence of autosomal dominant genetic variants; and absence of *TREM2* risk variants. In addition, the matched cohort was created based on age at death, sex, and varying *APOE* genotypes. Summary of donors’ pathologic, demographic, and genetic information can be found in Additional file 1: Table S2 with data on each individual donor in Additional file 1: Table S3.

#### Neuropathologic confirmation of bioinformatic results

6 um thick formalin fixed paraffin embedded slides of the middle frontal gyrus, angular gyrus, or superior/middle temporal gyrus from AD or neurologically normal brain donors were co-stained for either SOX9 or VIM and NFIA or ERBB4. Slides were deparaffinized in xylene and rehydrated using a descending ethanol series. Slides were microwaved for 15 min at 99 °C in citrate antigen retrieval solution (Vector Laboratories H-3300). Slides were washed in 0.1 M Tris buffer (pH 7.6) and blocked in 2% FBS/0.1 M Tris buffer (pH 7.6). 200 µL of primary antibody cocktail (monoclonal mouse-anti-SOX9 1:1000 Abcam ab76997; polyclonal chicken-anti-VIM 1:500 Novus NB300-223; polyclonal rabbit-anti-NFIA 1:50 Sigma HPA006111; polyclonal rabbit-anti-ERBB4 1:50 Sigma HPA012016) diluted in 2% FBS/0.1 M Tris buffer (pH 7.6) were added to each section. Slides were incubated in a humidified chamber overnight at 4 °C.

Antibodies were washed using 0.1 M Tris buffer (pH 7.6). Slides were blocked in 2% FBS/0.1 M Tris (pH 7.6), and 200 uL of secondary antibody cocktail (polyclonal goat-anti-mouse conjugated to AlexaFluor-488 1:500, Invitrogen A11029; goat-anti-chicken conjugated to AlexaFluor-488 1:500, Invitrogen A11039; goat-anti-rabbit conjugated to AlexaFluor-568 1:500, Invitrogen A11036) diluted in 2% FBS/0.1 M Tris (pH 7.6) were added to each section. Slides were incubated in a dark, humidified chamber at room temperature for 2–2.5 h. Antibodies were washed in 0.1 M Tris, and sections were stained with 300 nM DAPI in PBS.  Slides were washed 3× with PBS, and glass coverslips were placed on each slide with mounting media (ProLong Antifade Glass Mountant). Additional file 1: Table S3 includes which brain region was used for each set of stains for each brain donor.

#### Counting nuclei in immunofluorescence images

Each slide was visualized using a Leica TCS SPE laser scanning confocal microscope and imaged using a Leica DFC365 FX microscope camera. 15 step z-stacks of two random gray matter regions were imaged for each donor for each stain. DAPI was excited with a 405 nm laser and emission wavelengths 423–503 nm were captured; Alexa-Fluor 488 was excited with a 488 nm laser and emission wavelengths 500–590 nm were captured; Alexa-Fluor 568 was excited with a 561 nm laser and emission wavelengths 575–655 nm were captured; autofluorescence was measured by exciting the tissue with a 488 nm laser and capturing emitted wavelengths 600–776 nm. Microscope images were analyzed using Leica LAS X software. Max projections of each image were used to count total nuclei based on DAPI signal. Z-stacks were used to identify SOX9^+^, NFIA^+^, ERBB4^+^ and VIM^+^ nuclei/cells. Comparisons of proportions of SOX9^+^, NFIA^+^, ERBB4^+^, VIM^+^, and total nuclei were performed using Fisher’s Exact Tests from Prism 9 software.

## Results

### Identifying protoplasmic and fibrous astrocytes in an Alzheimer’s disease single nucleus RNA sequencing dataset

**Fig. 1:**
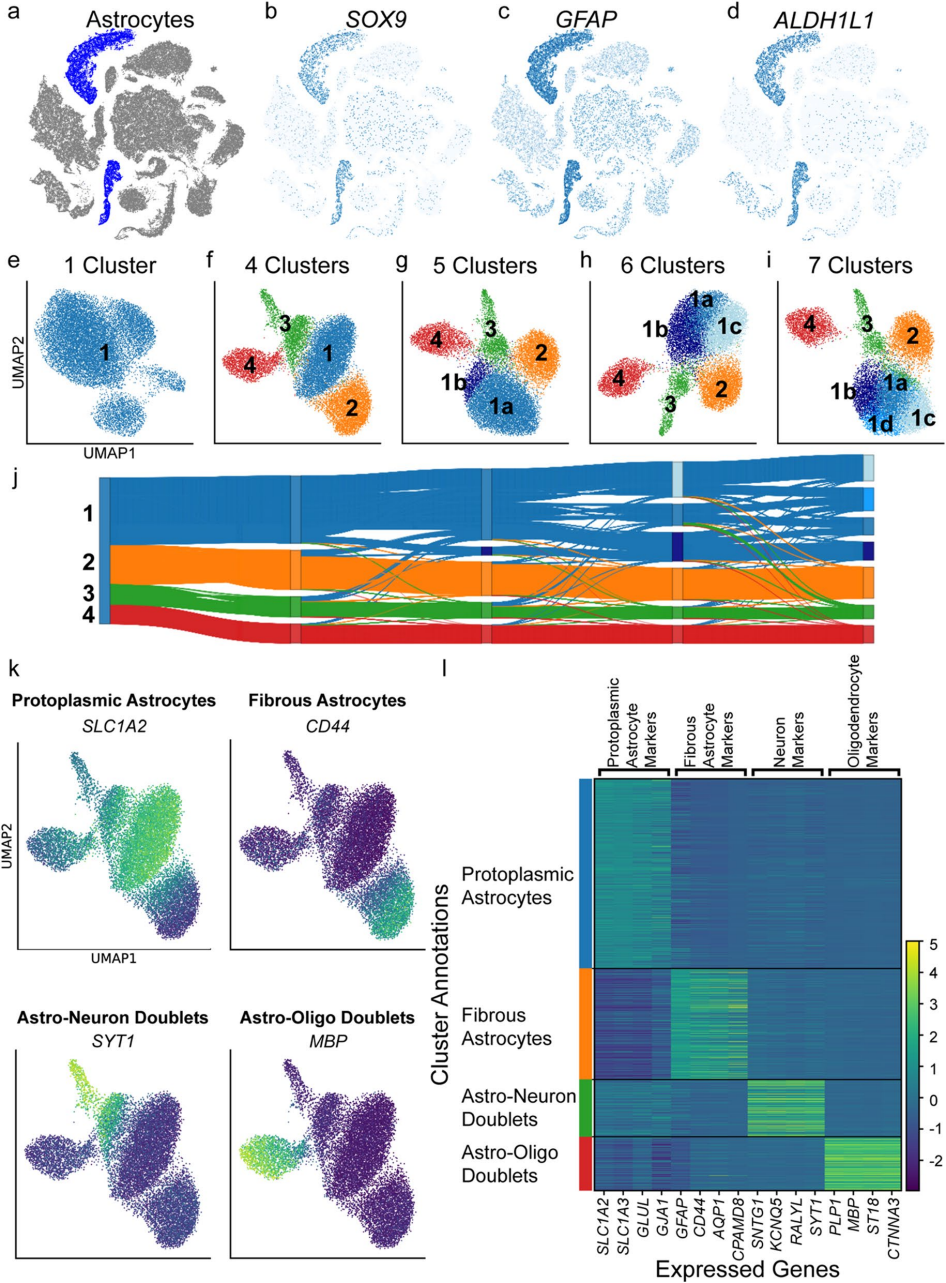
Identifying protoplasmic and fibrous astrocyte clusters. **a** t-distributed stochastic neighbor embedding (t-SNE) projection of 122,606 sequenced nuclei across 15 normal, pathologic aging, and Alzheimer’s disease human brains from a previously published single-nucleus RNA sequencing dataset [50] with astrocyte nuclei colored in blue. **b**–**d** Expression feature plots of known astrocyte-specific marker genes. **e**–**i** Uniform Manifold Approximation and Projection (UMAP) plots and **j** Sankey diagram of the 15,529 astrocyte nuclei as they were clustered at varying resolutions. **k** Expression feature plots of known marker genes for protoplasmic astrocytes, fibrous astrocytes, neurons, and oligodendrocytes at the four cluster resolution. **l** Heatmap showing cluster annotations based on differential gene expression

A single-nucleus RNA sequencing dataset encompassing normal, pathological aging, and Alzheimer’s disease brains [50] was reanalyzed here. The cluster containing astrocyte nuclei was identified based on expression of astrocyte-specific marker genes *SOX9*, *GFAP*, and *ALDH1L1* (Fig. 1a–d) and extracted for further analysis. These 15,529 astrocyte nuclei were re-clustered to varying resolutions using count adapted regularized Deep Embedded Clustering (CarDEC) [37], a deep learning algorithm that iteratively clusters nuclei and denoises gene expression data to enable accurate clustering and robust downstream analyses. At the one cluster resolution, astrocyte nuclei were visually split into four distinct groups (Fig. 1e). When clustered from four to seven clusters, the astrocytes continued to separate into four distinct groups with additional clusters being formed via subdividing the blue cluster (labeled “1”, Fig. 1f–i). A Sankey diagram of the astrocyte cluster assignments across clustering resolutions demonstrated the astrocyte nuclei stably adhered to one of four groups: blue/1, orange/2, green/3, and red/4 (Fig. 1j) with increased clustering resulting in the blue/1 group subdividing into smaller 1a, 1b, 1c, and 1d clusters. Clusters were composed of nuclei from all samples (Additional file 1: Fig. S1).

The four astrocyte clusters were annotated according to their differential gene expression patterns. The blue/1 cluster was enriched for genes associated with glutamate uptake and metabolism (*SLC1A2/EAAT2/GLT-1*, *SLC1A3/EAAT1/GLAST-1*, *GLUL*) as well as gap junction proteins (*GJA1*) that are most highly expressed in protoplasmic, gray matter astrocytes (Fig. 1k, l) [35, 70]. The orange/2 cluster showed an enrichment for genes (*GFAP*, *CD44, AQP1, CPAMD8*) highly expressed by fibrous, white matter astrocytes, interlaminar astrocytes, perivascular astrocytes, and subpial astrocytes [26, 35, 45, 57, 64]. White matter astrocytes are the most common of these populations and were likely to compose a majority of the cluster. The green/3 and red/4 clusters were enriched for neuron- and oligodendrocyte-specific genes, respectively, and likely resulted from the artifactual sequencing two nuclei (doublet) within the same droplet. These doublet instances were excluded from further downstream analysis.

### Spectrum of reactivity in gray matter astrocytes of Alzheimer’s disease brains

**Fig. 2:**
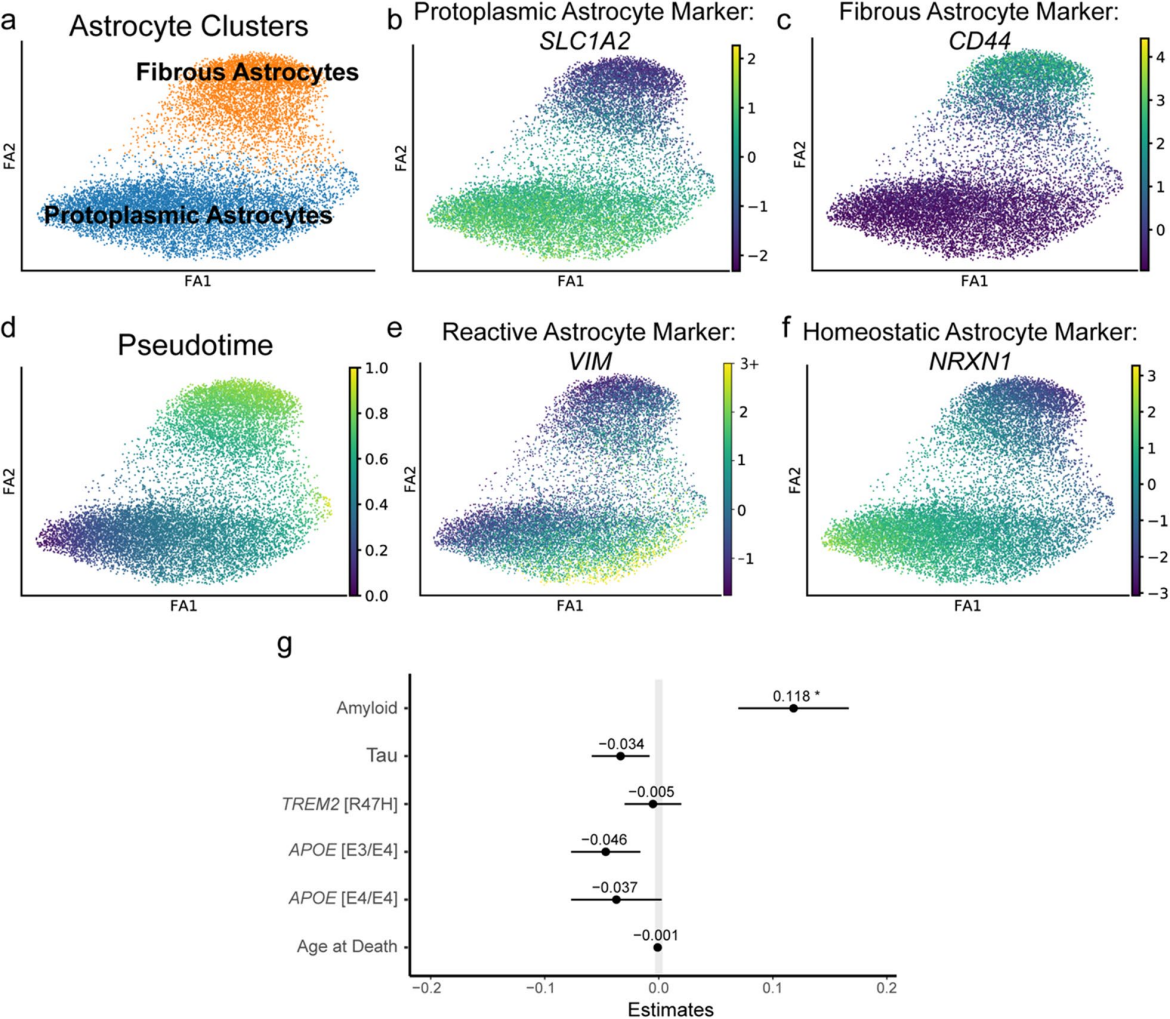
Protoplasmic astrocytes exist within a spectrum of reactivity. **a** Protoplasmic and fibrous astrocyte nuclei plotted using partition-based graph abstraction (PAGA). **b** *SLC1A2* expression in protoplasmic astrocytes. **c** *CD44* expression in fibrous astrocytes. **d** RNA trajectory analysis was used to calculate pseudotime values for astrocyte nuclei. **e** *VIM*, a known reactive astrocyte gene, increased along pseudotime. **f** *NRXN1*, a known homeostatic astrocyte gene, decreased along pseudotime. **g** Plot of linear mixed effects model coefficients for modeling nuclei pseudotime as a function of donors’ pathologic, genetic, and demographic features (*p = 0.040). Error bars represent standard error

Protoplasmic and fibrous astrocyte nuclei were re-plotted using PAGA (partition-based graph abstraction) (Fig. 2a–c) and analyzed using RNA trajectory analysis, which estimates a pseudotime value that reflects the degree of transcriptomic change from a starting nucleus. This pseudotime analysis is based on a more linear (i.e. topology preserving) visualization of the data in contrast with typical clustering algorithms. Pseudotime revealed distinct transcriptomic differences between protoplasmic and fibrous astrocytes (Fig. 2d). Protoplasmic astrocytes exhibited a spectrum of change over pseudotime, while fibrous astrocytes appeared homogeneous. Known reactive astrocyte genes, such as *VIM* [25], increased along protoplasmic astrocyte pseudotime (Fig. 2e), while known homeostatic astrocyte genes, such as *NRXN1* [27, 69], decreased along pseudotime (Fig. 2f), consistent with graded amounts of reactivity among protoplasmic astrocytes.

We hypothesized that protoplasmic astrocyte pseudotime in our analysis corresponded to the molecular changes associated with astrocyte reactivity. To explore what pathologic, genetic, and demographic factors correlated with pseudotime among protoplasmic astrocytes, we performed a linear mixed effects analysis using amyloid pathology, tau pathology, *APOE* allelic genotype, *TREM2 * R47H genotype, and age at death as co-variates and found that only amyloid pathology was significantly associated with an increase in pseudotime (Fig. 2g, amyloid pathology coefficient = 0.118, *p*-value = 0.040; tau pathology coefficient = − 0.034, *p*-value = 0.222; *TREM2* R47H coefficient = − 0.005, *p*-value = 0.842; *APOE* E3/E4 coefficient = − 0.046, *p*-value = 0.163; *APOE* E4/E4 coefficient = − 0.037, *p*-value = 0.376; age at death coefficient = − 0.001, *p*-value = 0.379). A linear mixed effects analysis was also performed for fibrous astrocytes using the same co-variates, and no factors significantly correlated with nuclei pseudotime (amyloid pathology coefficient = − 0.098, *p*-value = 0.052; tau pathology coefficient = 0.048, *p*-value = 0.065; *TREM2* R47H coefficient = 0.017, *p*-value = 0.473; *APOE* E3/E4 coefficient = 0.039, *p*-value = 0.183; *APOE* E4/E4 coefficient = 0.056, *p*-value = 0.152; age at death coefficient = − 0.001, *p*-value = 0.383). Overall, protoplasmic astrocyte pseudotime was observed to positively correlate with the presence of amyloid pathology, which is consistent with the idea that protoplasmic astrocytes are more reactive in brains containing amyloid pathology.

### Reactive astrocytes are characterized by downregulation of homeostatic genes

**Fig. 3:**
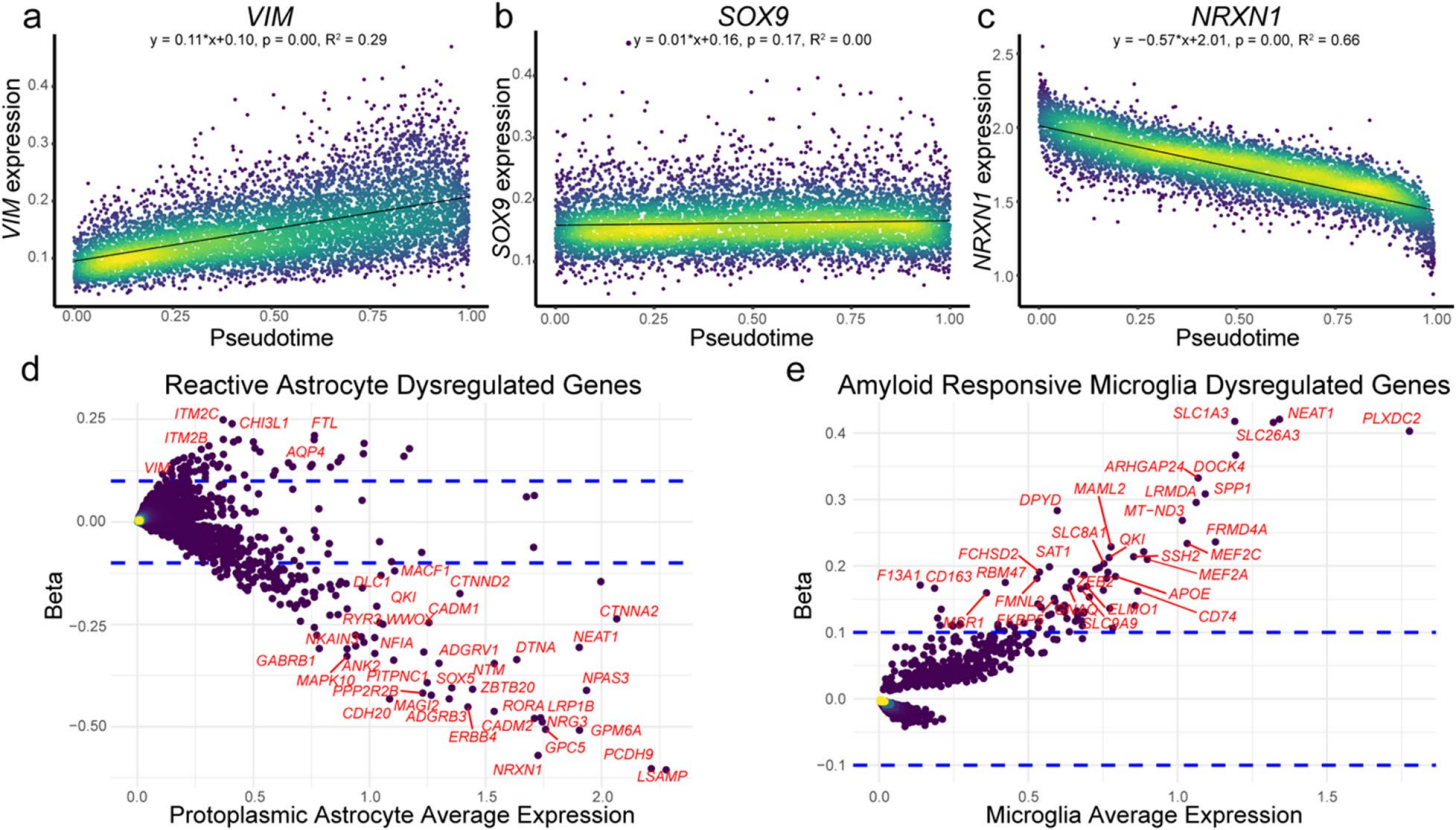
Reactive astrocytes exhibit pathologic transcriptome dysregulation. **a** Linear regressions of gene expression versus pseudotime were used to identify genes associated with astrocyte reactivity. Plots of gene expression as a function of pseudotime for **a** *VIM*, a reactive astrocyte gene, **b** *SOX9*, an astrocyte marker gene, and **c** *NRXN1*, a homeostatic astrocyte gene. Plots were colored by density of nuclei at each coordinate. **d** MA-style plot of dysregulated genes in reactive astrocytes and colored by density of genes at each coordinate. Genes’ average expression in protoplasmic astrocytes was plotted versus their pseudotime linear regression beta, a measure of effect size. Dotted blue lines represent threshold for linear regression beta used to identify significantly dysregulated genes. **e** MA-style plot of dysregulated genes in amyloid responsive microglia (ARMs) [50] and colored by density of genes at each coordinate. Dotted blue lines represent beta threshold used to identify significantly dysregulated genes. For **d**, **e**, plots include genes with non-zero betas (linear regression *p* < 0.05 after Bonferroni correction for multiple comparisons)

As pseudotime appeared to be a measure of astrocyte reactivity, the transcriptome-wide alterations associated with reactive astrocytes was assessed using linear regressions of protoplasmic astrocyte pseudotime versus gene expression for each of 17,012 expressed genes in order to determine how each gene’s expression increased or decreased as a function of pseudotime. Importantly, each linear regression calculated a beta (effect size), R^2^ (variance), and a *p*-value (significance) for each gene’s expression change in reactive astrocytes. We thresholded genes by Bonferroni-corrected *p*-value, beta, and R^2^ to identify 196 genes that changed the most over pseudotime and explained the most variance in pseudotime (Additional file 1: Fig. S2a). Of these 196 most dysregulated genes in reactive astrocytes, 52 genes, including *VIM*, increased over pseudotime (Fig. 3a). *SOX9*, whose protein product confers astrocyte identity exhibited no change over pseudotime (Fig. 3b). 144 genes, including *NRXN1*, decreased over pseudotime (Fig. 3c). To visualize global transcriptomic changes in reactive astrocytes, we generated an MA-style plot wherein each gene was plotted by its average expression in protoplasmic astrocytes versus its linear regression beta. This revealed that astrocyte reactivity appears to be characterized by a marked downregulation of homeostatic genes (*NRXN1*, *NRG3*, *GPC5*, *ERBB4*) in addition to a modest upregulation of genes canonically associated with astrocyte reactivity (*VIM, CHI3L1*) (Fig. 3d).

As a control for our analytic methodology, we repeated this analysis on 2773 microglia nuclei from the same single nucleus RNA sequencing dataset and found amyloid responsive microglia were predominantly characterized by an upregulation in gene expression (Fig. 3e).

**Fig. 4:**
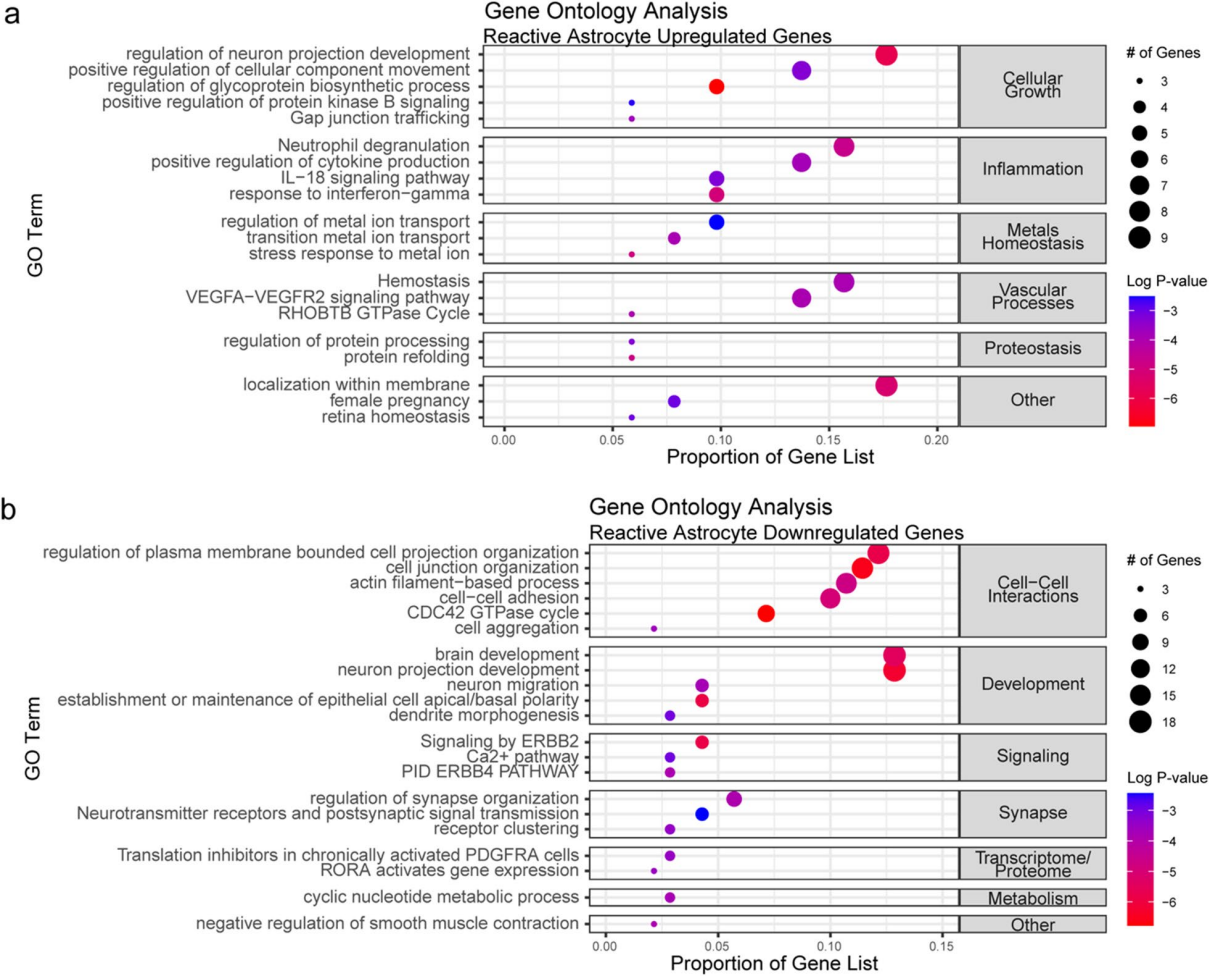
**a** Gene ontology analyses of 52 top upregulated genes and **b** 144 top downregulated genes identified by thresholding for genes’ linear regression beta (effect size), R^2^ (variance), and Bonferroni-corrected p-value (significance) (Additional file 1: Fig. S2)

Gene ontology analyses were conducted to identify biological pathways affected by reactive astrocyte transcriptomic dysregulation. Upregulated genes were associated with terms associated with reactive astrocytosis including cellular growth, inflammation, metal homeostasis, vascular processes, and proteostasis (Fig. 4a). Downregulated genes were associated with terms that reflect the normal homeostatic function of protoplasmic astrocytes including cell–cell interactions, development, ERBB signaling, synapse regulation, and transcriptome/proteome regulation (Fig. 4b).

### Comparing human reactive astrocyte transcriptome with rodent reactive astrocyte changes and AD GWAS hits

Our human reactive astrocyte transcriptome was compared to transcriptomic changes observed in astrocytes from Alzheimer’s disease animal models. Single nucleus RNA sequencing was recently performed on the 5xFAD mouse model, which develops severe amyloid pathology and astrogliosis [29, 51]. A cluster of astrocytes were found to be enriched among 5xFAD mice, and they exhibited dysregulation of 224 genes that were expressed in our human astrocyte dataset (Additional file 2: Table S4). 29 dysregulated genes were shared with our human reactive astrocytes (Additional file 1: Fig. S3a). Gene enrichment analysis revealed that the 5xFAD disease associated astrocyte signature was enriched among the 196 genes most dysregulated in our human reactive astrocyte transcriptome (*p* < 0.0001, odds ratio = 14.97). Shared genes were predominantly upregulated, suggesting that reactive astrocytes from the 5xFAD animal model recapitulate portions of the upregulation observed in human reactive astrocytes but do not fully recapitulate the transcriptomic downregulation observed in human reactive astrocytes.

Translating-ribosome-affinity purification sequencing (TRAP-seq) was recently performed on the cortex of APP/PS1 mice, which develop amyloid pathology and astrogliosis [59]. Of 2499 dysregulated genes in APP/PS1 astrocytes (Additional file 2: Table S4), 41 were dysregulated in human reactive astrocytes, representing no statistical enrichment (*p* = 0.641, Odds ratio = 1.092) (Additional file 1: Fig. S3b). TRAP-seq was also performed on the cortex and spinal cord of the MAPT^P301S^ mouse model, which develop phospho-tau pathology and astrogliosis [79]. In the cortex, of 36 dysregulated genes, 0 were dysregulated in human reactive astrocytes. In the spinal cord, of 1686 dysregulated genes (Additional file 2: Table S4), 34 were shared with human reactive astrocytes, representing no statistical enrichment (*p* = 0.1322, odds ratio = 1.367) (Additional file 1: Fig. S3c). These results suggest that reactive astrocytes from AD animal models variably recapitulate human reactive astrocyte transcriptomic dysregulation and do not seem to appreciably demonstrate the transcriptomic downregulation observed in human reactive astrocytes.

Next, we assessed whether putative AD risk genes were dysregulated in reactive astrocytes. Of 77 genes implicated in AD disease development via recent genome wide association studies (GWAS) [3], 65 were expressed in astrocytes, none of which were among the 196 top dysregulated genes identified in the current study (Additional file 1: Fig. S4, Additional file 2: Table S4).

### Pathologic confirmation of homeostatic protein downregulation in reactive astrocytes

**Fig. 5:**
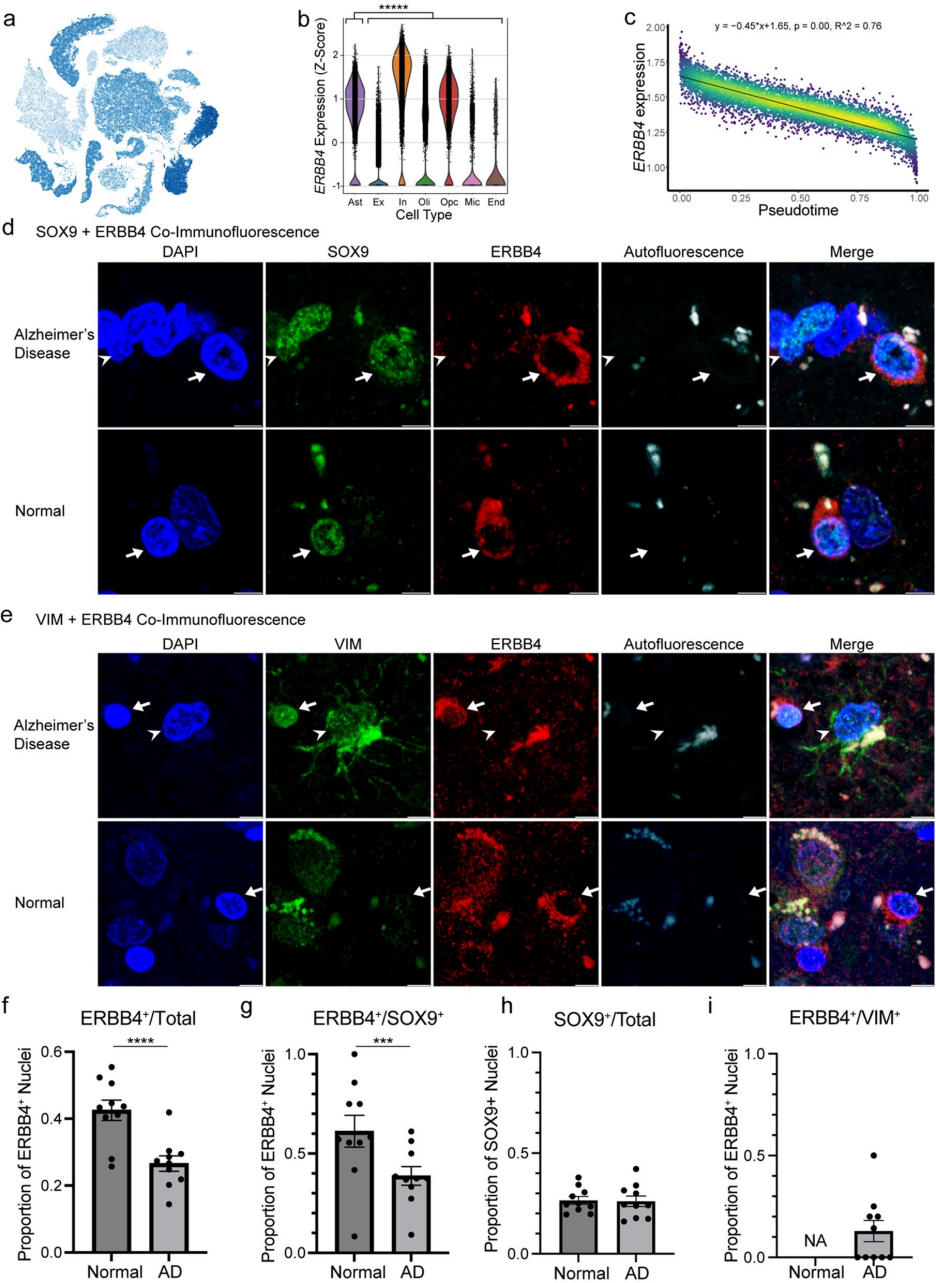
Pathologic confirmation of receptor tyrosine-protein kinase ERBB4 downregulation in reactive astrocytes. **a** Feature plot of *ERBB4* expression. **b** Violin plot of *ERBB4* expression across seven brain cell types (differential gene expression of astrocytes vs rest using Wilcoxon rank-sum test with Benjamini–Hochberg correction for multiple comparisons, *****p < 0.00001, 2.28 log-e fold change). *Ast* astrocyte, *Ex* excitatory neuron, *In* inhibitory neuron, *Oli* oligodendrocyte, *Opc* oligodendrocyte progenitor cell, *Mic* microglia, *End* endothelia. **c** Plot of *ERBB4* denoised, normalized, and natural log-transformed gene expression versus pseudotime with linear regression modeling (Bonferroni adjusted *p*-value). Colored by nuclei density at each coordinate. **d** Co-immunofluorescence images of AD (top) and normal (bottom) brain tissues stained for DAPI, SOX9, and ERBB4. Arrows point to DAPI^+^SOX9^+^ERBB4^+^ astrocyte nuclei; arrow heads point to DAPI^+^SOX9^+^ERBB4^−^ astrocyte nuclei. **e** Co-immunofluorescence images of AD (top) and normal (bottom) brain tissues stained for DAPI, VIM, and ERBB4. Arrows point to DAPI^+^VIM^−^ERBB4^+^ nuclei; arrow heads point to DAPI^+^VIM^+^ERBB4^−^ reactive astrocyte nuclei. All scale bars correspond to 5 um. **f** Proportions of ERBB4^+^ nuclei among total nuclei for 10 normal and 10 AD brains (Fisher’s exact test, ****p < 0.0001, odds ratio = 1.979). Aggregated count data from SOX9 + ERBB4 and VIM + ERBB4 co-immunofluorescence images. **g** Proportions of ERBB4^+^ nuclei among SOX9^+^ astrocyte nuclei for 10 normal and 10 AD brains (Fisher’s exact test, ***p = 0.0004, odds ratio = 2.472).** h** Proportions of SOX9^+^ nuclei among total nuclei for 10 normal and 10 AD brain co-stained for SOX9 and ERBB4 (Fisher’s exact test, *p* = 0.9422, odds ratio = 1.012). **i** Proportion of ERBB4^+^ nuclei among VIM^+^ reactive astrocytes for 10 normal and 10 AD brains. No reactive astrocytes were observed in images of normal brains. Error bars represents standard error of the mean (SEM)

*ERBB4* encodes the receptor tyrosine-protein kinase ErbB-4, a member of the epidermal growth factor receptor (EGFR) family, which is crucial for cellular signaling, differentiation, proliferation, and survival [75]. In the brain, *ERBB4* is significantly enriched in astrocytes but also expressed in other cell types throughout the brain (Fig. 5a, b). We found *ERBB4* to be transcriptionally downregulated in reactive astrocytes (Fig. 5c), and ERBB4 signaling also appeared as an enriched GO term among downregulated genes (Fig. 4b). To assess protein-level changes, we performed co-immunofluorescence imaging of ERBB4 and SOX9 (pan-astrocyte marker) as well as ERBB4 and VIM (reactive astrocyte marker) on normal and AD brains (summary characteristics in Additional file 1: Table S2, individual characteristics in Additional file 1: Table S3). We first counted the total number of ERBB4^+^ nuclei as a proportion of total nuclei observed in normal and AD donors and found a significantly decreased proportion of ERBB4^+^ nuclei in AD donors (27.4%) vs normal controls (41.6%) (Fig. 5f).

Next, to determine whether there was a downregulation of ERBB4 specifically in astrocytes, we assessed ERBB4 expression amongst SOX9^+^ astrocyte nuclei in AD and normal controls. In neurologically normal brains, SOX9^+^ astrocytes consistently expressed ERBB4. However, in AD brains, many SOX9^+^ astrocytes were often negative for ERBB4 (Fig. 5d). We counted the proportion of SOX9^+^ nuclei that were ERBB4^+^ in each donor and found a significantly decreased proportion of astrocytes that expressed ERBB4 (i.e. SOX9^+^ERBB4^+^ nuclei) among the AD donors (39.2%) compared to normal donors (60.3%) (Fig. 5g). The number of SOX9^+^ nuclei as a proportion of total DAPI^+^ nuclei did not differ between normal and AD brains (26.1% vs 25.9%) (Fig. 5h).

To visualize ERBB4 changes specifically in reactive astrocytes, we assessed ERBB4 expression amongst VIM^+^ reactive astrocytes in AD and normal controls. In AD brains, most VIM^+^ reactive astrocytes stained negative for ERBB4 (Fig. 5e), while VIM^+^ reactive astrocytes were not found in the normal brains. We counted the number of VIM^+^ reactive astrocytes expressing ERBB4 and found only 10.9% of reactive astrocytes stained positive for ERBB4 (Fig. 5i).

### Reactive astrocytes exhibit a downregulation of transcription factors

**Fig. 6:**
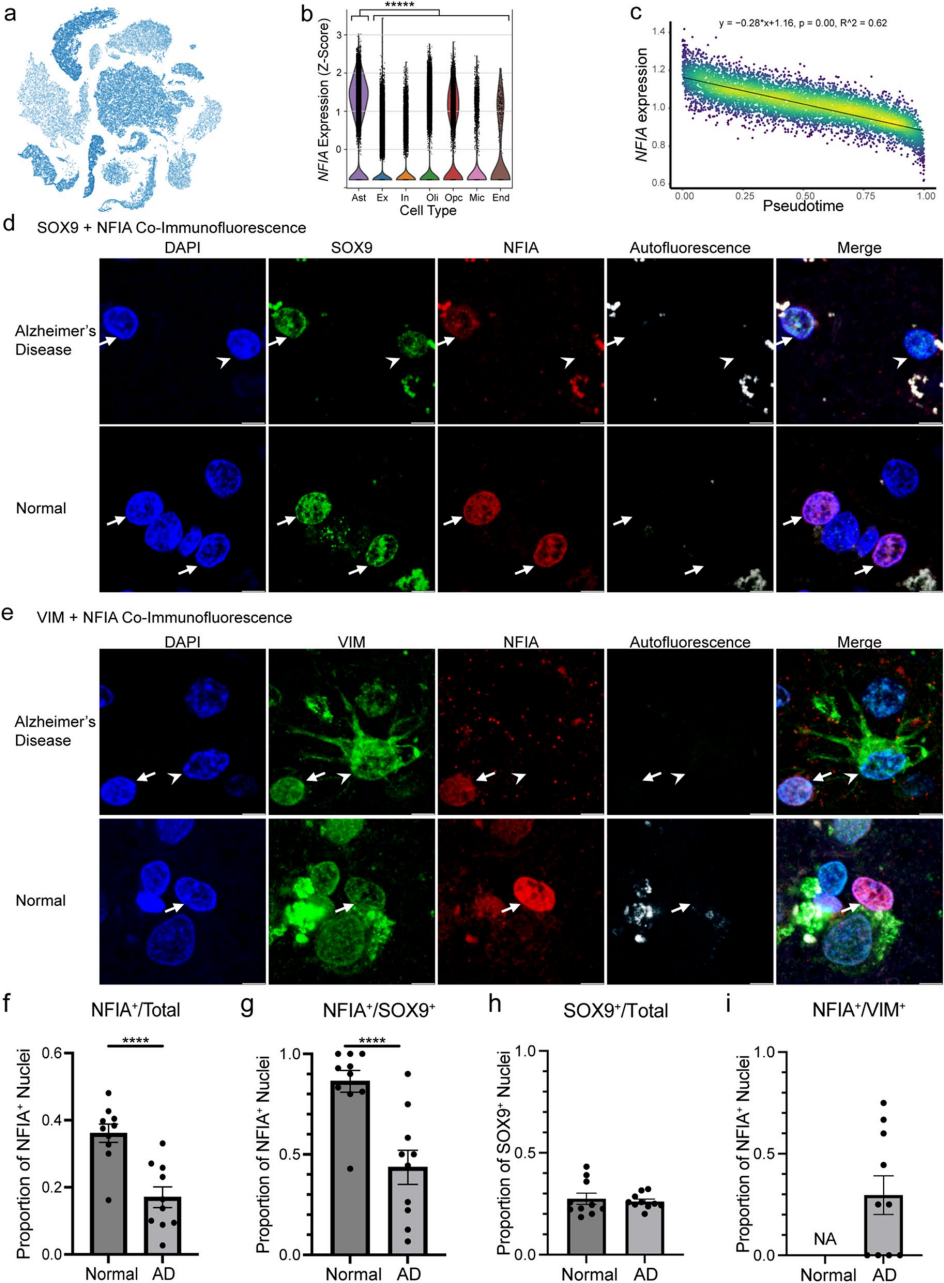
Pathologic confirmation of transcription factor NFIA downregulation in reactive astrocytes. **a** Feature plot of *NFIA* expression. **b** Violin plot of *NFIA* expression across seven brain cell types (differential gene expression of astrocytes vs rest using Wilcoxon rank-sum test with Benjamini–Hochberg correction for multiple comparisons, *****p < 0.00001, 2.53 log-e fold change). *Ast* astrocyte, *Ex* excitatory neuron, *In* inhibitory neuron, *Oli* oligodendrocyte, *Opc* oligodendrocyte progenitor cell, *Mic* microglia, *End* endothelia. **c** Plot of denoised, normalized, and natural log-transformed *NFIA* expression vs pseudotime with linear regression modeling (Bonferroni adjusted *p*-value). Colored by nuclei density at each coordinate. **d** Co-immunofluorescence images of AD (top) and normal (bottom) brain tissues stained for DAPI, SOX9, and NFIA. Arrows point to DAPI^+^SOX9^+^NFIA^+^ astrocyte nuclei; arrow heads point to DAPI^+^SOX9^+^NFIA^−^ astrocyte nuclei. **e** Co-immunofluorescence images of AD (top) and normal (bottom) brain tissues stained for DAPI, VIM, and NFIA. Arrows point to DAPI^+^VIM^−^NFIA^+^ nuclei; arrow heads point to DAPI^+^VIM^+^NFIA^−^ reactive astrocyte nuclei. All scale bars correspond to 5 um. **f** Proportions of NFIA^+^ nuclei among total nuclei for 10 normal and 10 AD brains (Fisher’s exact test, ****p < 0.0001, odds ratio = 2.739). Aggregated count data from SOX9 + NFIA and VIM + NFIA co-immunofluorescence images. **g** Proportions of NFIA^+^ nuclei among SOX9^+^ astrocyte nuclei for 10 normal and 10 AD brains (Fisher’s exact test, ****p < 0.0001, odds ratio = 9.910).** h** Proportions of SOX9^+^ nuclei among total nuclei for 10 normal and 10 AD brains co-stained for SOX9 and NFIA (Fisher’s exact test, *p* = 0.6395, odds ratio = 1.083). **i** Proportion of NFIA^+^ nuclei among VIM^+^ reactive astrocytes for 10 normal and 10 AD brains. No reactive astrocytes were observed in images of normal brains. Error bars represent standard error of the mean (SEM)

In addition to ERBB4 downregulation, we found transcription factors to be enriched among the 144 significantly downregulated genes (Additional file 1: Fig. S2b, *p* = 0.0134, odds ratio = 1.930). To confirm that reactive astrocytosis is associated with transcription factor downregulation, we assessed NFIA expression, a transcription factor that is significantly enriched in astrocytes (Fig. 6a, b) and is transcriptionally downregulated in reactive astrocytes (Fig. 6c). We performed co-immunofluorescence imaging of NFIA and SOX9 (pan-astrocyte marker) as well as NFIA and VIM (reactive astrocyte marker) on normal and AD brains. First, we counted the total number of NFIA^+^ nuclei as a proportion of total nuclei in normal and AD donors and found a significantly decreased proportion of NFIA^+^ nuclei in AD donors (16.9%) vs normal controls (37.5%) (Fig. 6f).

Next, we assessed NFIA expression amongst SOX9^+^ astrocyte nuclei, and we found neurologically normal brains showed consistent NFIA expression in SOX9^+^ astrocytes. However, in Alzheimer’s disease brains, many SOX9^+^ astrocytes stained negative for NFIA (Fig. 6d). We counted the number of NFIA^+^ nuclei amongst SOX9^+^ astrocytic nuclei in each donor and found a significantly decreased proportion of SOX9^+^NFIA^+^ nuclei among the AD donors (41.1%) compared to normal controls (87.4%) (Fig. 6g). Importantly, the proportion of SOX9^+^ nuclei amongst all nuclei did not differ between normal and AD brains (27.6% vs 26.1%) (Fig. 6h).

To directly visualize NFIA changes in reactive astrocytes, we assessed NFIA expression amongst VIM^+^ reactive astrocytes in AD and normal controls. In Alzheimer’s disease brains, most VIM^+^ reactive astrocytes stained negative for NFIA (Fig. 6e), while VIM^+^ reactive astrocytes were not found in the normal brains. Only 28.8% of VIM^+^ reactive astrocytes expressed NFIA (Fig. 6i).

## Discussion

Growing evidence implicates neuroinflammation, including reactive astrocytes, in Alzheimer’s disease pathogenesis [20, 40]. Normally, astrocytes tile the brain, enforce the blood brain barrier, maintain ion and pH homeostasis, and provide synaptic support with astrocytes enwrapping half of neocortical excitatory synapses and uptaking 80% of released glutamate [70]. However, in AD, there is a breakdown in these homeostatic functions with astrocytic endfeet retraction and blood brain barrier breakdown leading to infiltration of the brain by peripheral immune system cells and toxic blood components [67, 82]; furthermore, neurological cation imbalance, acidosis, and decreased astrocyte-mediated glutamate uptake contribute to excitotoxicity and neurodegeneration [17, 30, 72, 73, 84]. Morphologically, reactive astrocytes hypertrophy, increase expression of cytoskeletal proteins such as GFAP and vimentin [20, 25], and are found surrounding amyloid plaques where their processes extended into the plaque core [21, 23, 29, 30, 46]. When exposed to amyloid-beta peptides, cultured rodent astrocytes take on reactive morphologies, increase GFAP expression, and decrease glutamate uptake [48, 55]. Similarly, transgenic AD animal models concomitantly develop amyloid plaques and reactive astrocytes in close proximity [51, 52], suggesting astrocyte reactivity may be a response to pathological amyloid.

Our results suggest that astrocyte reactivity correlates with the presence of amyloid plaque pathology but not tau pathology. In patients, plasma GFAP is associated with CSF Aβ42/40 but not p-Tau181 [19]. Astrocytes tile the brain parenchyma, and their processes are equipped with numerous receptors for sensing the extracellular environment, including toll-like receptors (TLRs) and receptors for advanced glycoxidation end-products (RAGE) that can bind to amyloid beta aggregates [62, 70]. As a result, they may be more adept at responding to extracellular pathologic proteins, such as amyloid plaques, compared to intracellular protein aggregates. However, reactive astrocytes are features of diseases without protein aggregation, such as ischemic stroke. Reactive astrocytes are also found in neurodegenerative conditions without amyloid plaque pathology, such as Lewy body disease (LBD) and frontotemporal lobar degeneration (FTLD) with tau or TDP-43 inclusions [25]. Future studies are necessary to characterize the similarities and differences between human reactive astrocytes across neurological conditions.

To better understand reactive astrocytes’ pathological changes beyond GFAP upregulation, a variety of transcriptomic studies have been conducted. The earliest attempts to understand reactive astrocytes’ transcriptomic responses to pathologic insults led to A1 neurotoxic and A2 neuroprotective astrocyte designations in response to LPS stimulation and ischemia [13, 43, 44, 80]. Next, to understand astrocytes’ changes in aging and neurodegenerative contexts, astrocytes were isolated from AD mouse models and assessed using RNA sequencing and more recently, single-cell RNA sequencing, enabling the identification of differential astrocyte response patterns to acute injury and neurodegenerative conditions with some shared features (increased cytoskeleton, extracellular matrix, and pro-inflammatory cytokine and interferon signaling) [8, 16, 19, 24, 29, 32, 53]. However, rodent reactive astrocyte signatures have exhibited poor concordance with transcriptomic changes in humans assessed via bulk sequencing of human tissue or isolated astrocytes [16]. The development of single-nucleus RNA sequencing technologies has enabled more nuanced examinations into cell type heterogeneity in human tissue [28, 41, 47, 85].

By analyzing the astrocytes within an AD single nucleus RNA sequencing dataset using novel bioinformatic approaches, we found protoplasmic, gray matter astrocytes to exist within a spectrum of reactivity that is predominantly characterized by downregulation of homeostatic genes. We observed moderate upregulation of hypertrophy and inflammation-associated genes that have been canonically associated with reactive astrocytes, but the magnitude of change for these upregulates genes is minor compared to the downregulation of homeostatic genes. A recent consensus statement indicated that reactive astrocytes may undergo simultaneous loss of homeostatic function and gain of protective/detrimental functions [25]. Our findings support this idea with an emphasis on homeostatic gene downregulation dominating the transcriptomic changes in AD-associated reactive astrocytes. Due to the importance of astrocytes in maintaining brain homeostasis, the downregulation of homeostatic astrocyte genes raises the possibility that there is a loss of normal astrocyte function in AD which could potentially contribute to neurodegeneration. Further studies are needed to determine whether restoring reactive astrocyte homeostatic functions can promote an environment that is more conducive to neuronal health.

The highest upregulated gene in human reactive astrocytes was *ITM2C* (also known as *BRI3*). A similar gene, *ITM2B* (also known as *BRI2*) was also upregulated in the dataset. Interestingly, they encode secreted chaperone proteins that have been shown to decrease the rate of Aβ42 fibril formation in vitro and co-localize with amyloid-beta plaques in human AD tissue [22], suggesting that reactive astrocytes may increase secretion of these chaperone proteins to limit amyloid pathology. Moreover, astrocytes have been shown to phagocytose amyloid-beta peptides, suggesting that astrocytes play an active role in removing amyloid-beta from the extracellular environment [33, 63]. The second highest upregulated gene was *CHI3L1*, which is thought to regulate neuroinflammation and is currently being trialed as a marker of neuroinflammation [6, 15, 66]. The third highest upregulated gene was *FTL*, which encodes ferritin light chain, which acts to sequester iron. The brain accumulates iron over time, especially in neurodegenerative disease conditions, and reactive astrocytes may upregulate *FTL* to sequester excess iron and inhibit neuron death [81]. Thus, the most upregulated genes in reactive astrocytes may help limit amyloid fibril formation, regulate neuroinflammation, and sequester iron.

Interestingly, many genes (*LSAMP, PCDH9, NRXN1, CADM2, CDH20, MAGI2, NTM, ADGRL3, NEBL, CADM1, CNTN1, CTNNA2*, *PRKCA*, *LPP*, *MACF1*, *FLRT2*, *NCAM1*, *TENM2*) and several gene sets associated with cell–cell interactions were among the most downregulated genes in our reactive astrocytes. Cellular adhesion proteins, such as neurexins, are important for maintaining the integrity of the tripartite synapse [27, 65, 69], and downregulation of these adhesion proteins may hamper reactive astrocytes’ interactions with nearby cells and synapses, reducing their abilities to regulate extracellular ion balance, pH, and glutamate concentrations, potentially contributing to excitotoxicity. Moreover, cell adhesion proteins participate in intercellular signaling via activating focal adhesion kinases (FAK), which promotes neuronal survival [10]; downregulation of astrocytic cell adhesion proteins and loss of protective signaling may additionally contribute to neuron loss in Alzheimer’s disease.

Downregulation of epidermal growth factor receptor (EGFR) family members and signaling effectors was another robust finding in our reactive astrocyte transcriptome with 7 genes (*ERBB4*, *FYN*, *NRG3*, *WWOX*, *PRKCA*, *AKT3*, *ERBIN*) implicated. Physiological roles of ErbB2 and ErbB4 signaling in astrocytes is inconsistent between rodent and human studies. Cultured, mouse astrocytes with constitutively active EGFR family signaling resulted in increased proliferation, while cultured, human fetal astrocytes exhibited increased proliferation following ErbB4 knockdown [12, 61]. Nevertheless, given ERBB signaling’s important roles in proliferation and cell growth in other contexts [75] and combined with our results indicating ERBB4 and ERBB2 signaling pathway downregulation in human reactive astrocytes, further studies are needed to clarify the role of EGFR family signaling in human reactive astrocytes.

We found transcription factors, including NFIA, to be enriched among downregulated genes. NFIA, NFIB, and SOX6 have been shown to convert fibroblasts and stem cells into functional astrocytes, implicating these transcription factors in the establishment of astrocyte identity [9, 11, 58, 60, 68, 78]. Nfia knockout in astrocytes resulted in shortened processes and decreased morphological complexity within the cortex as well as decreased blood–brain-barrier function following white matter injury [31, 39]. Npas3 knockdown in astrocytes resulted in decreased synaptic densities in astrocyte-neuron co-cultures and animal models [42]. Forebrain-specific knockout of Zbtb20 resulted in an increase in GFAP^+^ astrocytes [49]. Furthermore, Rora^−/−^ astrocytes have been shown to exhibit increased inflammatory responses following IL-1-beta and TNF-alpha stimulations compared to WT astrocyte [34]. These results suggest that the downregulation of transcription factors may reflect a rewiring of the transcriptional program that maintains astrocyte homeostasis, reducing normal homeostatic astrocyte function.

Finally, age is the greatest risk factor for Alzheimer’s disease development, and aging has been shown to induce astrocyte senescence, a cellular state characterized by hypertrophy, secretion of pro-inflammatory proteins, and increased expression of GFAP and vimentin [14]. Astrocytes have been shown to take on a senescence phenotype following treatment with amyloid-beta oligomers [4, 18], and astrocytes expressing the senescent markers p16^INK4a^ and IL-6 have been found in the frontal cortex of aged patients, Alzheimer’s disease patients, and around amyloid plaques [4, 14]. However, there are large transcriptomic differences in senescent astrocytes induced by amyloid-beta oligomers versus aging, suggesting heterogeneity within astrocyte senescence [18]. We cannot rule out the possibility that the dysregulation observed in our reactive astrocyte transcriptome is in part associated with astrocyte senescence. However, senescence-associated markers were not upregulated in our dataset, suggesting our analysis captured transcriptomic changes in reactive astrocytes. Future studies will be necessary to unravel the differences between reactive and senescent astrocytes as well as their differential contributions to neurologic disease.

The limitations of this study should also be noted. Our analysis stemmed from a single dataset encompassing 11,337 astrocyte nuclei across 15 samples. Astrocytes are diverse, and it’s possible that additional homeostatic and reactive astrocyte phenotypes may exist in addition to the ones described here. Moreover, conclusions such as the association between the reactive astrocyte transcriptome identified here and the presence of beta-amyloid pathology requires more study and validation including the use of more quantitative measures of pathologic change in larger number of brain donors, together with emerging methods such as spatial transcriptomics. Also, all sequenced nuclei belonged to male donors, so sex-specific transcriptomic differences in reactive astrocytes were not able to be explored, and transcriptomic findings may not be generalizable to females. In addition, our assessments of reactive astrocytes’ phenotypic changes stemmed from sequencing nuclear RNAs. Although nuclear mRNA content has been found to correlate well with cytoplasmic mRNA content [2, 36], biases are also likely to be present. Cells ultimately rely on proteins to function, and although mRNA-level changes are likely to result in protein-level changes, this may not be true for all genes.

In summary, this study has identified an Alzheimer’s disease-associated human reactive astrocyte transcriptome that is most strongly characterized by downregulation of transcription factors and homeostatic genes. Due to the importance of astrocytes in maintaining brain homeostasis and providing neuronal support, this finding raises the possibility that reactive astrocyte downregulation of homeostatic function results in a loss of normal astrocyte function that may perhaps contribute to neurodegeneration.

## Supplementary Information

**Additional file 1: Table S1.** Single nucleus RNA sequencing cohort characteristics. **Table S2. **Validation cohort characteristics.** Table S3. **Individual validation case characteristics.** Fig S1.** Distribution of nuclei from each sample across cluster resolutions.** Fig S2.** **a** Dysregulated genes’ linear regression betas vs *R*^2^ values.** Fig S3. **Venn diagrams comparing transcriptomic dysregulation in human reactive astrocytes versus astrocytes from AD-relevant mouse models.** Fig. S4** **a** MA-style plots colored by AD GWAS hits.

**Additional file 2. Table S4.** Linear regressions of reactive astrocyte gene expression with analytical annotations.

## Data Availability

Our AD snRNAseq data will be deposited in GEO. Additional file 2: Table S4 includes the human reactive astrocyte transcriptome and its comparisons to datasets in this article.
