# Supplementary material for: Human Alzheimer’s disease reactive astrocytes exhibit a loss of homeostastic gene expression
Source: Acta Neuropathol Commun. 2023 Aug 2;11:127. doi: 10.1186/s40478-023-01624-8 (PMC10398957; doi:10.1186/s40478-023-01624-8)
Supplement: Supplementary file 1 — Additional file 1: Table S1. Single nucleus RNA sequencing cohort characteristics. Table S2. Validation cohort characteristics. Table S3. Individual validation case characteristics. Fig S1. Distribution of nuclei from each sample across cluster resolutions. Fig S2. a Dysregulated genes’ linear regression betas vs R2 values. Fig S3. Venn diagrams comparing transcriptomic dysregulation in human reactive astrocytes versus astrocytes from AD-relevant mouse models. Fig. S4 a MA-style plots colored by AD GWAS hits. [file 40478_2023_1624_MOESM1_ESM.docx]

| **Sample** | **Clinical Diagnosis(es)** | **Neuropathological Diagnosis(es)** | **AT score** | **A** | **B** | **C** | **Sex** | **Age at Death** | **PMI** | ***APOE* genotype** | ***TREM2* c.140G>A, p.Arg47His** |
| --- | --- | --- | --- | --- | --- | --- | --- | --- | --- | --- | --- |
| 1 | Normal | Unremarkable adult brain | A-T- | 0 | 0 | 0 | Male | 70 | 36 | E3/E3 | No |
| 2 | Normal | Primary age-related tauopathy (PART) | A-T- | 0 | 1 | 0 | Male | 59 | 17 | E3/E3 | No |
| 3 | Normal | Low ADNC | A+T- | 2 | 1 | 2 | Male | 84 | 23 | E3/E3 | No |
| 4 | Normal | Low ADNC, Cerebrovascular disease | A+T- | 1 | 0 | 0 | Male | 57 | 14 | E3/E4 | No |
| 5 | Possible Alzheimer's disease, Cerebrovascular disease | Intermediate ADNC, Limbic-predominant age-related TDP-43 Encephalopathy (LATE) | A+T- | 1 | 2 | 2 | Male | 88 | 17 | E3/E4 | No |
| 6 | Mild cognitive impairment | Intermediate ADNC | A+T- | 3 | 2 | 2 | Male | 85 | 8 | E3/E4 | No |
| 7 | Frontotemporal dementia-NOS | High ADNC, Limbic-predominant age-related TDP-43 Encephalopathy (LATE) | A+T- | 3 | 3 | 3 | Male | 78 | 18 | E3/E4 | Yes |
| 8 | Probable Alzheimer's disease | High ADNC, Limbic-predominant age-related TDP-43 Encephalopathy (LATE), Lewy body disease (amygdala predominant) | A+T+ | 3 | 2 | 3 | Male | 85 | 16 | E3/E3 | No |
| 9 | Possible Alzheimer's disease, Cerebrovascular disease | High ADNC, Limbic-predominant age-related TDP-43 Encephalopathy (LATE) | A+T+ | 3 | 3 | 3 | Male | 82 | 9 | E3/E3 | Yes |
| 10 | Probable Alzheimer's disease | High ADNC, Limbic-predominant age-related TDP-43 Encephalopathy (LATE), Lewy body disease (amygdala predominant) | A+T+ | 3 | 3 | 3 | Male | 86 | 6.5 | E3/E4 | No |
| 11 | Primary progressive aphasia, Logopenic variant | High ADNC | A+T+ | 3 | 3 | 3 | Male | 60 | 12 | E3/E4 | Yes |
| 12 | Probable Alzheimer's disease | High ADNC, Lewy body disease (amygdala predominant) | A+T+ | 3 | 3 | 3 | Male | 71 | 4.5 | E3/E4 | No |
| 13 | Probable Alzheimer's disease | High ADNC | A+T+ | 3 | 3 | 3 | Male | 54 | 18 | E3/E4 | No |
| 14 | Probable Alzheimer's disease | High ADNC | A+T+ | 3 | 3 | 3 | Male | 74 | 18 | E4/E4 | No |
| 15 | Probable Alzheimer's disease | High ADNC, Lewy body disease (amygdala predominant) | A+T+ | 3 | 3 | 3 | Male | 61 | 11 | E4/E4 | Yes |

**Table S1**. Individual clinical, neuropathologic, demographic, and genetic characteristics of the 15 normal, pathological aging, and Alzheimer’s disease brain donors used for single nucleus RNA sequencing experiment. PMI = Post-mortem interval.

| Characteristic |  | Neurologically Normal | Alzheimer's Disease | p-value |  |
| --- | --- | --- | --- | --- | --- |
| N |  | 10 | 10 |  |  |
| ADNC | A score | 0.90 (0.10) | 3.00 (0.00) |  |  |
|  | B score | 0.90 (0.18) | 3.00 (0.00) |  |  |
|  | C score | 0.10 (0.10) | 2.90 (0.10) |  |  |
| Age at Death, years |  | 72.90 (2.93) | 74.10 (2.05) | 0.741^a^ |  |
| Post-mortem interval, hours |  | 15.65 (1.94) | 14.45 (1.73) | 0.725^a^ |  |
| Sex, N | Male | 6 | 5 | 0.6531^b^ |  |
|  | Female | 4 | 5 |  |  |
| *APOE* Genotype | E2/E3 | 1 | 0 | 0.1705^b^ |  |
|  | E3/E3 | 7 | 4 |  |  |
|  | E3/E4 | 2 | 3 |  |  |
|  | E4/E4 | 0 | 3 |  |  |

**Table S2.** Summary Alzheimer’s disease neuropathologic change (ADNC), demographic, and genetic statistics of 10 neurologically normal and 10 AD brain donors used for pathologic confirmation co-immunofluorescence studies.

Data represent mean (SEM) unless otherwise noted.

^a^ Two-tailed t-test

^b^ Chi squared test

| Normal vs AD | Clinical Diagnosis(es) | Neuropathological Diagnosis(es) | A | B | C | Sex | Age at death | Post-mortem interval | *APOE* genotype | IF region |
| --- | --- | --- | --- | --- | --- | --- | --- | --- | --- | --- |
| Normal | Normal | Primary age-related tauopathy (PART) | 0 | 1 | 0 | Male | 67 | 26 | E3/E3 | MFG |
| Normal | Normal | Low ADNC | 1 | 1 | 0 | Female | 65 | 22 | E2/E3 | ANG |
| Normal | Normal | Low ADNC | 1 | 1 | 0 | Female | 75 | 18 | E3/E3 | SMT |
| Normal | Normal | Low ADNC | 1 | 1 | 0 | Male | 70 | 10.5 | E3/E3 | SMT |
| Normal | Normal | Low ADNC | 1 | 0 | 1 | Male | 71 | 13 | E3/E3 | MFG |
| Normal | Normal | Low ADNC | 1 | 0 | 0 | Female | 65 | 19 | E3/E3 | MFG |
| Normal | Normal | Low ADNC | 1 | 1 | 0 | Female | 67 | 11 | E3/E3 | SMT |
| Normal | Normal, alcoholism | Low ADNC | 1 | 1 | 0 | Male | 70 | 19 | E3/E3 | MFG |
| Normal | Depression | Low ADNC | 1 | 2 | 0 | Male | 89 | 12 | E3/E4 | ANG |
| Normal | Normal | Low ADNC, Lewy body disease (brainstem predominant) | 1 | 1 | 0 | Male | 90 | 6 | E3/E4 | ANG |
| AD | Probable Alzheimer's disease | High ADNC, Limbic-predominant Age-related TDP-43 Encephalopathy (LATE) | 3 | 3 | 3 | Female | 73 | 22 | E3/E3 | SMT |
| AD | Probable Alzheimer's disease | High ADNC, Lewy body disease (amygdala predominant), Limbic-predominant Age-related TDP-43 Encephalopathy (LATE) | 3 | 3 | 2 | Male | 69 | 7.5 | E4/E4 | ANG |
| AD | Probable Alzheimer's disease | High ADNC, Limbic-predominant Age-related TDP-43 Encephalopathy (LATE) | 3 | 3 | 3 | Female | 83 | 21 | E3/E4 | MFG/SMT |
| AD | Probable Alzheimer's disease | High ADNC, Lewy body disease (amygdala-predominant) | 3 | 3 | 3 | Male | 75 | 19 | E4/E4 | MFG |
| AD | Probable Alzheimer's disease | High ADNC | 3 | 3 | 3 | Male | 67 | 6.5 | E3/E4 | MFG |
| AD | Probable Alzheimer's disease | High ADNC, Limbic-predominant Age-related TDP-43 Encephalopathy (LATE), Lewy body disease (amygdala predominant) | 3 | 3 | 3 | Male | 68 | 5 | E3/E3 | ANG |
| AD | Probable Alzheimer's disease | High ADNC, Lewy body disease (amygdala predominant), Cerebrovascular disease | 3 | 3 | 3 | Female | 70 | 3.5 | E4/E4 | SMT |
| AD | Probable Alzheimer's disease | High ADNC | 3 | 3 | 3 | Female | 74 | 29 | E3/E4 | SMT |
| AD | Probable Alzheimer's disease | High ADNC, Lewy body disease (amygdala predominant) | 3 | 3 | 3 | Female | 75 | 12 | E3/E3 | MFG |
| AD | Probable Alzheimer's disease | High ADNC, Cerebrovascular disease | 3 | 3 | 3 | Male | 87 | 19 | E3/E3 | ANG |

**Table S3.** Individual clinical, neuropathologic, demographic, and genetic characteristics of the 10 normal and 10 Alzheimer’s disease brain donors used for co-immunofluorescence experiments.


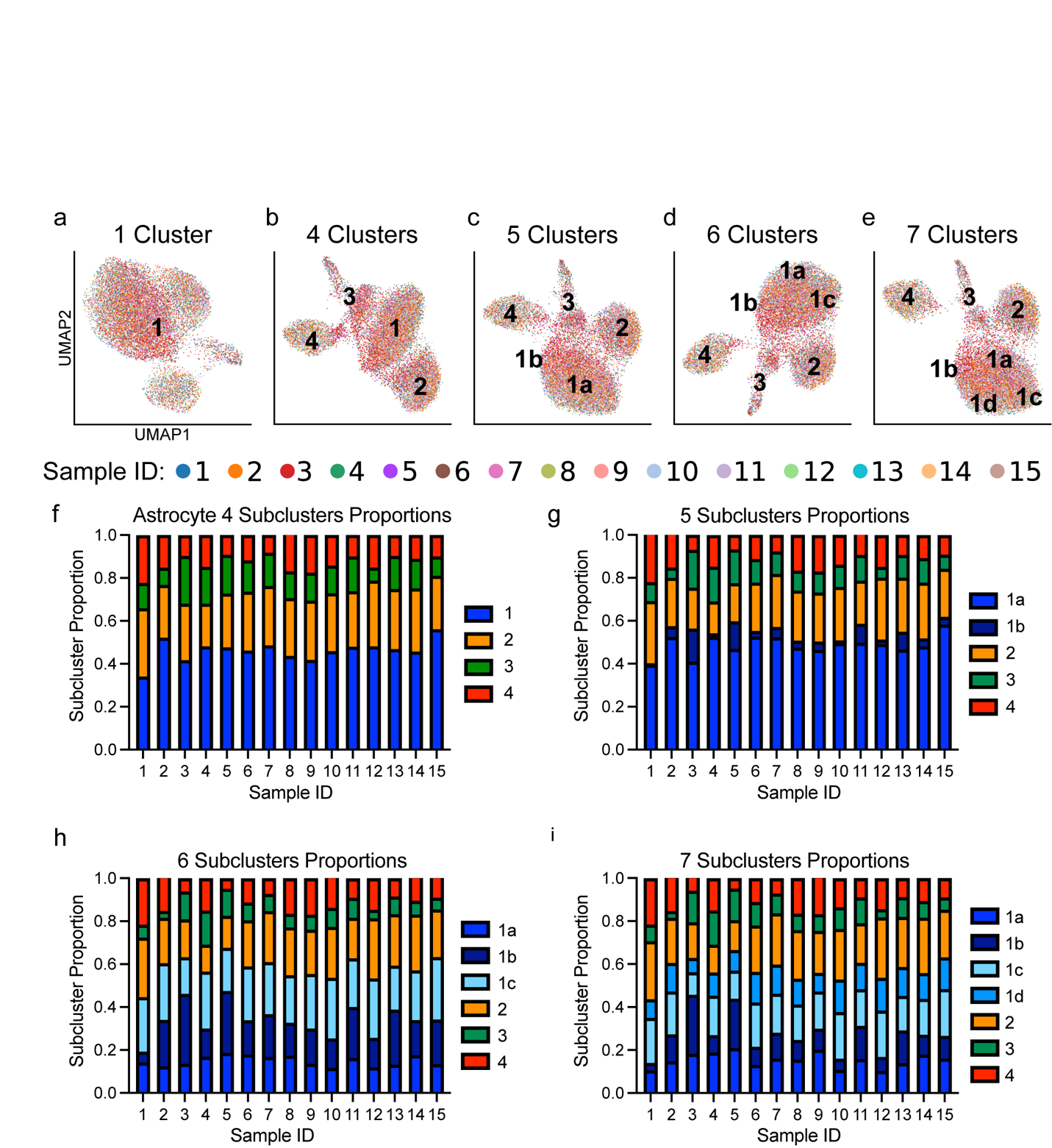


**Fig S1**. Distribution of nuclei from each sample across cluster resolutions. **a-e** UMAPs of astrocyte nuclei colored by sample origin across clustering resolutions. **f-I** Proportions of astrocyte nuclei assigned to each subcluster for each sample across clustering resolutions.


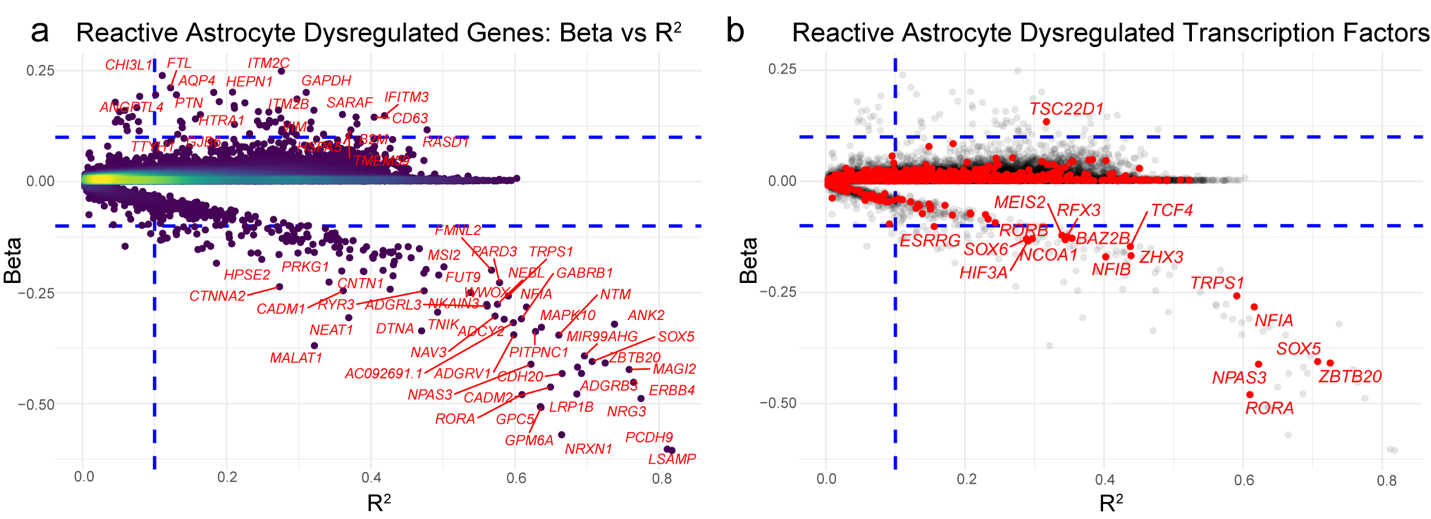


**Fig S2**. **a** Plotting dysregulated genes’ linear regression betas vs R^2^ values and thresholding (dotted lines) beta and R^2^ at 0.1 to identify genes that are most dysregulated across pseudotime and whose expression changes explain the most variance in pseudotime. Colored by density of genes at each coordinate. **b** Plotting dysregulated genes’ linear regression betas vs R^2^ values with transcription factors colored in red. Transcription factors were enriched amongst thresholded downregulated genes (Chi-squared test, p = 0.0134, odds ratio = 1.930). Plots include genes with non-zero betas (linear regression p < 0.05 after Bonferroni correction for multiple comparisons).

**
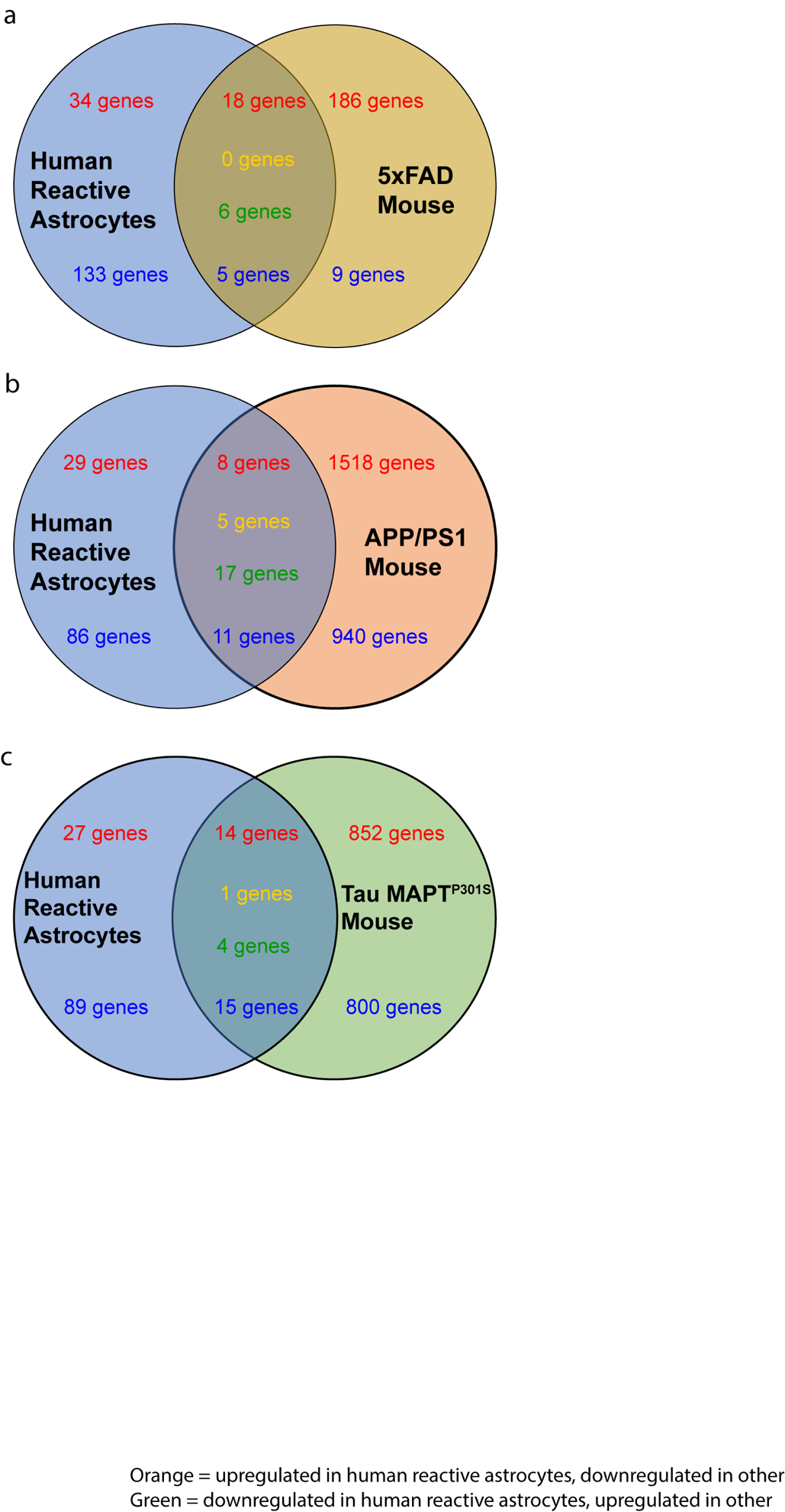
**

**Fig S3.** Venn diagrams comparing transcriptomic dysregulation in human reactive astrocytes versus astrocytes from AD-relevant mouse models: **a** 5xFAD mice [29], **b** APP/PS1 mice [32], and **c** Tau MAPT^P301S^ mice [32]**.** Red lettering represents upregulated genes and blue lettering represents downregulated genes. Orange lettering represents genes upregulated in human reactive astrocytes and downregulated in mouse models. Green lettering represents genes downregulated in human reactive astrocytes and upregulated in mouse models.

**
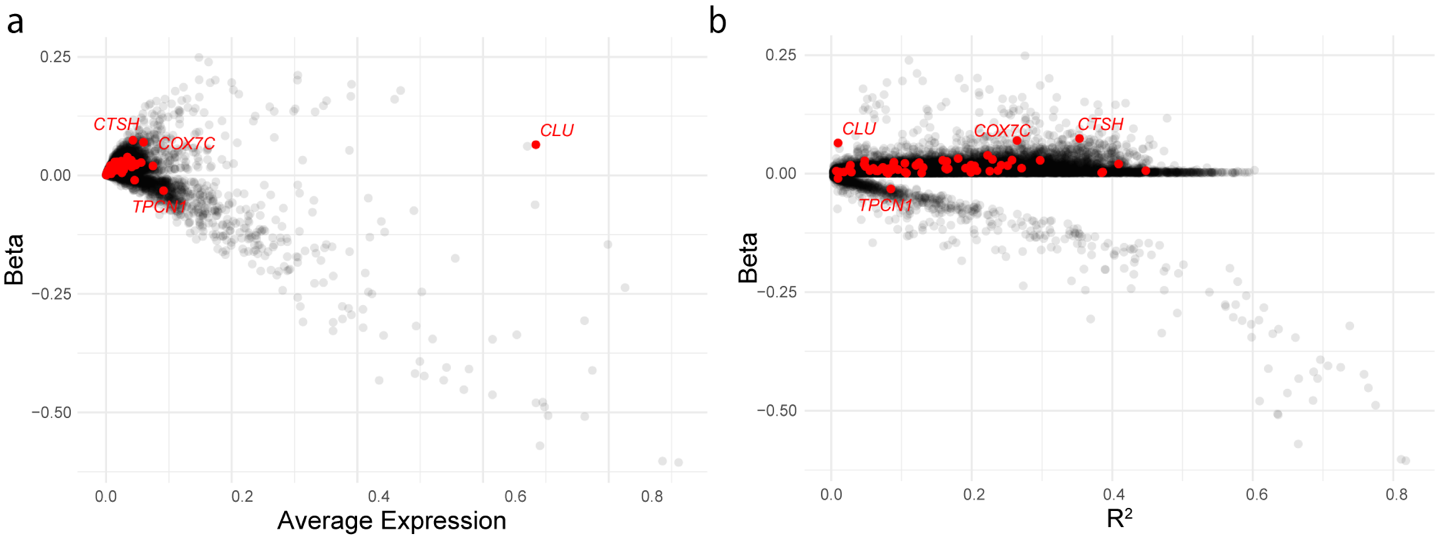
**

**Fig. S4 a** MA-style plot and **b** linear regression beta vs R^2^ plot of human reactive astrocyte dysregulated transcriptome colored by AD GWAS hits [3]. Plots include genes with non-zero betas (linear regression p < 0.05 after Bonferroni correction for multiple comparisons).
